# Regularity of bedtime, wake-up time, and time in bed in mid-life: associations with cardiometabolic health markers with adjustment for physical activity and sedentary time

**DOI:** 10.1186/s44167-023-00040-6

**Published:** 2024-01-05

**Authors:** Laura Nauha, Vahid Farrahi, Heidi Jurvelin, Timo Jämsä, Maisa Niemelä, Leena Ala-Mursula, Maarit Kangas, Raija Korpelainen

**Affiliations:** 1 Research Unit of Population Health, University of Oulu, 5000, 90014 Oulu, Finland; 2 Research Unit of Health Sciences and Technology, University of Oulu, 5000, 90014 Oulu, Finland; 3 Institute for Sport and Sport Science, TU Dortmund University, Dortmund, Germany; 4 Northern Ostrobothnia Hospital District, Kajaanintie 50, 90220 Oulu, Finland; 5 Medical Research Center, Oulu University Hospital and University of Oulu, 5000, 90014 Oulu, Finland; 6 Infrastructure for Population Studies, Northern Finland Birth Cohorts, Faculty of Medicine, University of Oulu, 5000, 90014 Oulu, Finland; 7 Department of Sports and Exercise Medicine, Oulu Deaconess Institute Foundation Sr., 365, 90100 Oulu, Finland

**Keywords:** Sleep–wake -rhythm, 24-h activity rhythm, Sleep regularity, Sleep consistency, Physical activity, Sedentary time, Chronotype, Circadian health

## Abstract

**Background:**

Insufficient sleep has been linked to the accumulation of cardiometabolic risks while physical activity acts as a protective factor. Also, sleep regularity may play a critical role in maintaining optimal cardiometabolic health. This cross-sectional study examined the association between device-based sleep regularity, waking activity behaviors, and cardiometabolic health markers, including blood pressure level; abdominal adiposity level; and blood glucose, insulin, and cholesterol.

**Methods:**

We included 3698 members of the Northern Finland Birth cohort 1966 who participated in the follow-up study at the age of 46 years between 2012 and 2014 (women 61%). We used seven-day standard deviations of device-based bedtime, wake-up time, and time in bed to reflect sleep regularities. As covariates in linear regression models, we used commonly known potential risk factors in (gender, education, marital status, work schedule, smoking status, alcohol risk use, seven-day time in bed mean, chronotype). In addition to the previous, we used either sedentary time or total physical activity as a covariate (B coefficients with 95% confidence intervals CI).

**Results:**

When we considered sedentary time with other covariates, irregularities in bedtime, wake-up time, and time in bed were associated with unfavorable cardiometabolic health markers, such as higher body mass index (bedtime regularity: 0.194, 95% CI [0.072, 0.316], p = 0.002); higher diastolic blood pressure levels (time in bed regularity: 0.175, 95% CI [0.044, 0.306], p = 0.009); and higher 2-h glucose levels (wake-up time regularity: 0.107, 95% CI [0.030, 0.184], p = 0.006). When we considered total physical activity with other covariates, only irregular bedtime was associated with higher waist circumference (B 0.199, 95% CI [0.042, 0.356], p = 0.013). Irregularities in bedtime and wake-up time were not associated with higher diastolic blood pressure, higher visceral fat area or higher fasting insulin level after considering sedentary time or total physical activity with other covariates (in all, p > 0.05).

**Conclusions:**

In middle-aged, physical activity appears to weaken the adverse relationship between irregular sleep and cardiometabolic health markers, although the interpretation of the impact of sedentary time remains less conclusive. The clinical significance and extent of the observed associations warrant further investigation.

**Supplementary Information:**

The online version contains supplementary material available at 10.1186/s44167-023-00040-6.

## Background

Adequate sleep plays a key role in preventing cardiometabolic problems and diseases, such as diabetes, hypertension, heart disease, and stroke [1, 2]. However, adequate sleep is not only actual hours slept, and factors such as sleep regularity have been shown to be associated with cardiometabolic health markers [3, 4]. The sleep–wake rhythm is an approximately 24-h biological rhythm that is controlled by the suprachiasmatic nuclei of the hypothalamus [5]. Sleep regulation has been proposed to be the interplay between sleep–wake homeostasis and an internal circadian rhythm that repeats itself approximately every 24 h [6, 7].

An individual’s chronotype is reflected in the variations in circadian preferences regarding daily sleep, wake, and high alertness times. Chronotype differences are based on the different phases of an internal circadian rhythm (i.e., a morning-type individual’s circadian rhythm runs earlier, while an evening-type individual’s rhythm runs later regarding the surrounding time of day [8]. A recent population-based study found that chronotype, and especially the evening chronotype, was associated with higher cardiometabolic risk [9]. Moreover, Reutrakul et al. [10] found evidence of the associations between evening-type individuals and several cardiometabolic disorders and unhealthy behaviors.

The internal circadian rhythm can become disrupted, which eventually leads to misalignment or internal desynchronization. Behind potential circadian disruption can be work schedules, irregular sleeping habits, chronotype or meal timing [11]. This loss of coordination of circadian rhythm can have negative consequences for sleep–wake cycles and numerous other biological functions [12–14]. Previous studies have suggested that a misalignment between circadian rhythm and sleep time is associated with metabolic risk factors that predispose individuals to cardiometabolic issues, such as greater body mass indexes and waist circumferences, higher fasting glucose and blood pressure levels, and lower HDL cholesterol levels. [15] Most of the evidence of the association between the abovementioned misalignment and cardiometabolic health has come from studies of shift workers [10, 16] with only a few studies of the general population having been conducted [15, 17, 18]. Based on the existing literature, a deeper understanding of the role of daily sleep regularity in the health of the general population is required to inform public health guidelines and future research [3, 4]. As knowledge about the 24-h sleep–wake homeostasis process and its links to health increases, recommendations for improved quality of sleep could also be required to include a recommendation for sleep time and regularity.

Recent studies suggest that sleep and movement intensities that make up the 24-h day are codependent [19–21]. Physical activity (PA) is associated with improved quality of sleep [22]. There is evidence that higher levels of physical activity may offset all-cause mortality risks associated with short sleep duration [23]. Overall, studies have shown that daytime activity facilitates circadian alignment [24, 25]. A survey-based prospective cohort study of middle-aged adults showed that engaging in moderate- to high-intensity PA in the daytime is effective in preventing insomnia [26]. In this context, it is also important to consider the convincing research evidence indicating that more PA is associated with better cardiometabolic health [27]. Therefore, when studying the association between sleep habits and cardiometabolic health, waking activity behaviors should also be considered [28].

In sleep-related studies, time in bed (TIB) refers to the duration of the primary sleep period, including wakefulness occurring before, during and after the major sleep episode [29, 30]. Bedtime refers to the time at which TIB starts, and wake-up time refers to the point at which TIB ends. Sleep–wake -rhythm regularity across multiple days can be calculated from the information of sleep timings, such as bedtime or sleep duration by calculating standard deviation (SD) of the variable under consideration between measured days [17, 18, 31].

The aim of this population-based study is to examine the association between sleep regularity, waking activity behaviors, and cardiometabolic health markers among middle-aged Northern Finland Birth Cohort 1966 (NFBC1966) participants. As an indicator of sleep regularity, we use device-based bedtime, wake-up time, and time in bed (TIB) regularities.

## Methods

### Participants

The study comprised data from the Northern Finland Birth Cohort (NFBC1966), a longitudinal birth cohort including all Oulu and Lapland newborns whose births were expected in 1966 (N = 10,331) [32, 33]. The cohort members were regularly monitored prospectively through a wide range of clinical measurements, interviews, and postal questionnaires. This cross-sectional study included members of the NFBC1966 who participated in the most recent follow-up study at the age of 46 years (between 2012 and 2014) and agreed to wear accelerometer-based activity monitors to measure daily activity. The data collected in the 46-year follow-up study included self-reported data on the individuals’ health behaviors (n = 7146 [69.2%]) and whether they attended a clinical examination (n = 5832 [56.5%]).

### Measurements

#### Accelerometer

Participants attending the clinical examinations were asked to wear accelerometer-based activity monitors (Polar Active, Polar Electro Oy, Kempele, Finland) [34] continuously over 24 h, and while sleeping, for 14 consecutive days on their nondominant wrists. In this study, only seven consecutive days of data was included for each participant. Polar Active outputs metabolic equivalents (METs) every 30 s using background information (body height, body weight, age, and sex)) [35]. The intensity levels produced by Polar Active have been shown to be more comparable between different accelerometer-based methods than the commonly used limits defined by a hip accelerometer (e.g., sedentary time ≤ 1.5 METs, low PA 1.5–3 METs and moderate exercise 3–6 METs). For example, using the limits set by the ActiGraph (model GT3X), the Polar Active’s < 2 MET threshold was found to give similar results to the ActiGraph’s < 100 movements per minute limit [36]. In addition, Polar Active has been shown to detect well energy expenditure during free-living (correlation coefficient 0.88) and during training protocol including strength training activities for lower and upper body and cycling (correlation coefficient 0.79) [34]. Any recorded day with more than 2.5 h of constant activity with low MET values (< 1 MET [interpreted as nonwear time]) during the 24-h timeframes was considered invalid.

#### Bedtime, wake-up time, and time in bed regularities

Time in bed was identified from Polar Active MET values using our in-laboratory-validated algorithm. The algorithm identified all the potential sleep periods within each 24-h timeframe of 18:00:00 to 17:59:30. The longest sleep period was considered the TIB. The used algorithm proved accurate when determining the sleep times of a sample of young adults from a representative population [37]. For the present study, we verified the method through a visual assessment. Three researchers visually estimated bedtimes and wake-up times from accelerometry data from 150 randomly selected subjects [50 records per researcher]. On average, visually estimated bedtimes and wake-up times differed from the algorithm’s corresponding times by less than 16 min.

Sleep regularity was quantified from accelerometry data by the seven-day standard deviations of bedtime, wake-up time, and time in bed (TIB). Participants providing seven consecutive days’ worth of valid accelerometry data were included in this study (N = 3698) to better estimate sleep behaviors on both workdays and days off [38, 39].

#### Sedentary time and physical activity

Sedentary time and PA during waking hours were calculated using the Polar Active accelerometry data for each day. Daily means for sedentary time and total PA were calculated for each participant. Sedentary time [(min/day)] included all time with a recorded intensity of between 1 and 1.99 METs. Total PA, including all activities with a recorded intensity of 2 METs or higher, was calculated by multiplying each MET value by its duration (total PA [MET min/day]) [40, 41]. PA intensity level thresholds were based on the thresholds used by the manufacturer [42].

#### Cardiometabolic health markers

After fasting for 12 h overnight and abstaining from smoking and drinking coffee, participants attended a clinical examination. Trained nurses measured the participants’ heights, weights, and waist circumferences (WC), and the participants’ body mass indexes [BMI (kg/m^2^)] were calculated. After a period of restful sitting (at least 10 min), blood pressure (BP) was measured using the upper area of each participant’s right arm using an Omron M10-IT automatic blood pressure monitor (Omron M3, Omron Healthcare Europe BV, Netherlands). The blood pressure [systolic blood pressure (SBP) and diastolic blood pressure (DBP)] represented the mean of the three consecutive readings. Plasma glucose, serum insulin, total cholesterol, high-density lipoprotein (HDL) cholesterol, low-density lipoprotein (LDL) cholesterol, and triglyceride levels were analyzed using fasting blood samples [43, 44]. Participants who had not previously been diagnosed with either type 1 or type 2 diabetes were also asked to undergo an oral glucose tolerance test during which the plasma insulin and glucose levels were measured at 75 g. Two-hour post load plasma insulin and glucose levels were obtained from the results of the oral glucose tolerance test. Body fat (%), fat mass (kg), and visceral fat area (cm^2^) were estimated using bioimpedance measurements (InBody720, InBody, Seoul, Korea).

#### Covariates

Based on the question of work schedule, the participants were divided into three groups: day work, shift work, and not working/no information available. We used the shortened morningness–eveningness questionnaire (MEQ) to determine the participants’ chronotypes and classified participants into morning-type, day-type, and evening-type individuals [41, 45]. Scores on the shortened MEQ range from 5 to 27, and the sum score is grouped into three chronotypes: 5–12 as evening-type, 13–18 as day-type, and 19–27 as morning-type [46]. Participants self-reported their smoking status (non-smoker or former smoker, current smoker), alcohol consumption (g/day), education level, and marital status. Information concerning the participants’ current use of medication for hypertension, diabetes, hyperlipidemia, and central nervous system-related issues (e.g., antipsychotics) was gathered from the questionnaires. Heavy alcohol drinkers were defined according to the alcohol consumption instructions of the Finnish Institute for Health and Welfare, which sets the level for men at ≥ 40 g/day and the level for women at ≥ 20 g/day [47].

#### Statistical analyses

**Fig. 1:**
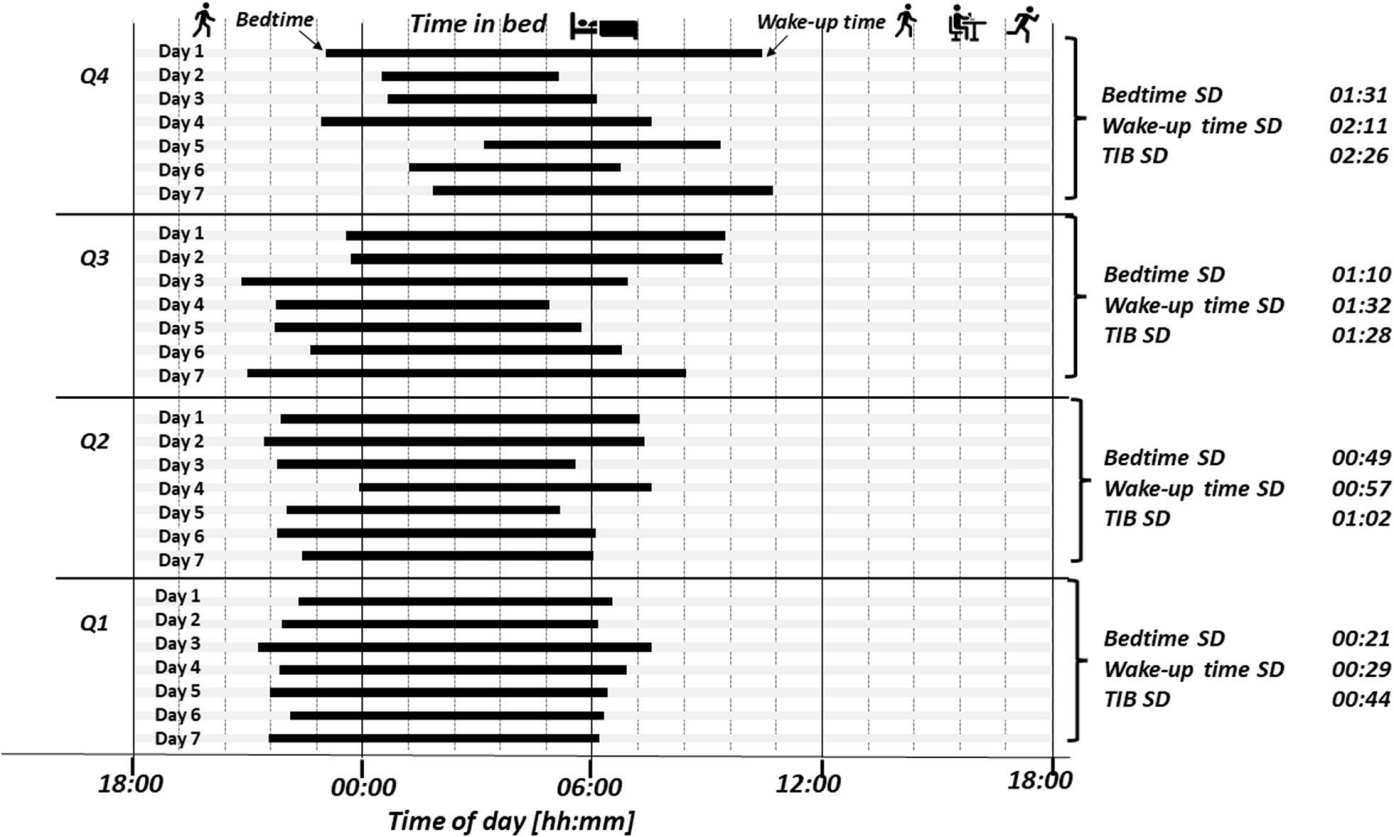
Sleep regularity quartiles showing the increasing standard deviations (increasing irregularities) of bedtime, wake-up time, and time in bed

Bedtime, wake-up time, and TIB schedule regularities were recoded to four equally sized groups and labeled as quartiles [one to four (Q1–Q4)] and characteristics of the participants (the distributions of sociodemographic factors, lifestyle factors, sleep-related factors, and cardiometabolic health markers) were calculated for each quartile. Figure 1 illustrates four examples of various sleep–wake -rhythm based on the quartiles (Q1–Q4) of bedtime, wake-up time, and TIB regularities showing the increasing standard deviations (increasing irregularities) of bedtime, wake-up time, and time in bed from quartile 1 to quartile 4. For example, the four quartiles for bedtime regularity were a seven-day standard deviation of bedtime of ≤ 00:39:52 (Q1), a seven-day standard deviation of bedtime of between 00:39:53 and 00:58:50 (Q2), a seven-day standard deviation of bedtime of between 00:58:51 and 01:29:20 (Q3), and a seven-day standard deviation of bedtime of ≥ 01:29:21 (Q4). The statistical significance of the group differences was analyzed using chi-square tests for normally distributed data and Kruskal–Wallis tests for skewed data with Tukey’s pairwise comparisons. The Bonferroni post-hoc comparison was applied to compare the quartiles. Sensitivity analysis was performed to determine whether results remained stable when all participants who reported any shift work were omitted from analyses.

Multivariate linear regression analysis was conducted to analyze the associations between bedtime, wake-up time, and TIB regularities and cardiometabolic health markers. There is evidence to suggest that some of the variables describing sleep rhythmicity and duration may have U-shaped relationship with markers of cardiometabolic health. For example, although irregular sleep rhythm has been linked to health problems [15], extremely regular sleep rhythm is not recommended in clinical sleep treatments because of stress management [48, 49]. Accordingly, we examined U-shaped associations between bedtime regularity, wake-up time regularity, or TIB regularity and cardiometabolic health markers, and stratified the analysis when there was evidence for a U-shaped relationship. To examine the U-shaped relationship, the variable and quadratic of the variable were added to the regression model, and the significance of the quadratic variable was checked. If evidence for a U-shaped relationship between bedtime regularity, wake-up time regularity, or TIB regularity and cardiometabolic health markers was observed (i.e., a quadratic component remained significant), the analysis for that outcome was stratified using 1 h as the cut point (≤ 01:00:00 and > 01:00:00). The cut point was defined as approximately 1 standard deviation difference from the average of bedtime, wake-up time or TIB regularity. Scatterplots with a quadratic curve between cardiometabolic health markers and exposures are available in Additional file (Additional file 1). Based on previous literature, we tested the associations with three incremental models. Model 1 was adjusted for gender, education, marital status, work schedule, smoking status, alcohol risk use, time in bed, chronotype and medication use (blood pressure/diabetes/lipids/medication affecting the central nervous system). Moreover, Model 1 was further adjusted for sedentary time (Model 2A), or physical activity (Model 2B). Bedtime, wake-up time and TIB regularity variables and cardiometabolic health markers underwent a process of log transformation before inclusion in the regression analyses to fulfill normality assumption. Linear regression models met all assumptions: we checked homoscedasticity from the scatterplots of residuals; multicollinearity using VIF (variance inflation factor) values with a score of below 10 indicating that the assumption is met; and normality of the residuals was checked from the normal probability plot. Statistical significance was set at p < 0.05. All analysis was performed using IBM SPSS Statistics (Version 25.0, IBM Corporation).

## Results

The average bedtime of the 3,698 participants over seven consecutive nights (N = 25,886 nights) was 23:21:43 (SD = 01:50:33), and the average wake-up time was 07:17:13 (SD = 01:49:06). More than half of the study population was women (men 39%; women 61%). There were more morning-type individuals (41%) than evening-type individuals (6%), but most of the study participants were day-type individuals (53%). On average, time in bed (TIB) was 07:56:31 (SD = 01:42:40). Participants in Q1 were the most regular sleepers, while participants in Q4 were the most irregular sleepers. Tables 1, 2, and 3 provide all the descriptive characteristics of the study population across the quartiles (Q1–Q4) of bedtime, wake-up time, and TIB regularities. The characteristics of the 3698 middle-aged birth cohort participants according to midpoint of sleep regularity are presented in Additional file (Additional file 2: Table S1). In addition, a correlation matrix to describe the correlations between the bedtime, waketime, time in bed, and midpoint of sleep regularity values is presented in Additional file (Additional file 3: Table S2). Overall, there was significant positive correlation among these variables ranging between 0.42 and 0.79, with highest correlation coefficient observed between midpoint of sleep regularity and bedtime regularity (0.79), followed by midpoint of sleep regularity and bedtime regularity (0.78).

**Table 1:** Characteristics of the 3698 middle-aged birth cohort participants according to the quartiles of bedtime regularity (standard deviations of accelerometer-measured bedtime over seven days)

	Bedtime regularity (hh:mm:ss)				
	Q1 ≤ 00:39:52	Q2 00:39:53–00:58:50	Q3 00:58:51–01:29:20	Q4 ≥ 01:29:21	*p-value*
*n* (total = 3698)	925	924	925	924	
Factors related to sleep and time in bed					
Bedtime (hh:mm)	23:02 (00:53)	23:08 (00:58)	23:17 (01:09)	23:57 (01:34)	** < 0.001**
Wake-up time (hh:mm)	07:01 (00:59)	07:12 (01:01)	07:17 (01:07)	07:39 (01:25)	** < 0.001**
Time in bed (hh:mm)	07:59 (00:51)	08:04 (00:50)	08:00 (01:00)	07:42 (01:09)	** < 0.001**
Chronotype (*n* [%])					** < 0.001**
Morning-type	399 (44.9)	356 (40.5)	353 (40.4)	357 (41.3)	> 0.05
Day-type	453 (51)	486 (55.4)	471 (53.9)	432 (49.9)	> 0.05
Evening-type	36 (4.1)	36 (4.1)	50 (5.7)	76 (8.8)	** < 0.05**
Work schedule (*n* [%])					** < 0.001**
Day work	658 (71.1)	646 (69.9)	626 (67.7)	497 (53.8)	** < 0.05**
Shift work	108 (11.7)	129 (14.0)	137 (14.8)	235 (25.4)	** < 0.05**
Not working/no information available	159 (17.2)	149 (16.1)	162 (17.5)	192 (20.8)	> 0.05
Sociodemographic and lifestyle factors					
Men (*n* [%])	316 (34.2)	319 (34.6)	374 (40.6)	440 (47.7)	** < 0.001**
High education (*n* [%])	280 (31.8)	266 (30.9)	255 (29.8)	196 (23.0)	** < 0.001**
Current smoker (*n* [%])	93 (10.4)	125 (14.1)	170 (19.5)	232 (26.7)	** < 0.001**
Heavy alcohol drinkers* (*n* [%])	42 (4.5)	60 (6.5)	68 (7.4)	104 (11.3)	** < 0.001**
Total PA (MET min/day)	1159.2 (362.2)	1094.4 (300.3)	1054.9 (331.0)	1038.9 (333.5)	** < 0.001**
Sedentary time (min)	566.5 (93.8)	571.0 (85.7)	581.9 (87.7)	588.6 (89.7)	** < 0.001**
Cardiometabolic health markers					
WC (cm)	88.3 (12.6)	89.7 (13.2)	91.5 (13.7)	93.4 (13.9)	** < 0.001**
Men	95.1 (10.6)	96.0 (11.6)	97.5 (12.6)	98.6 (12.7)	**0.006**
Women	84.8 (12.2)	86.3 (12.8)	87.4 (13.0)	88.6 (13.3)	** < 0.001**
BMI (kg/m^2^)	26.0 (4.7)	26.4 (4.8)	27.0 (5)	27.2 (5)	** < 0.001**
SBP (mmHg)	123.8 (15.7)	124.1 (15.3)	124.8 (15.3)	126.3 (16.2)	**0.044**
DBP (mmHg)	83.5 (10.3)	83.7 (10.9)	84.6 (10.1)	85.6 (10.7)	**0.001**
Body fat (%)	28.6 (9.0)	29.2 (9.2)	29.6 (9.5)	29.2 (9.6)	0.203
Fat mass (kg)	21.7 (9.9)	22.7 (10.7)	23.8 (11.3)	23.9 (11.3)	** < 0.001**
Visceral fat area (cm^2^)	98.8 (39.1)	102.6 (40.9)	107.7 (43.2)	108.7 (42.9)	** < 0.001**
Fasting insulin (pmol/L)	8.98 (6.08)	9.38 (9.13)	9.62 (6.5)	10.45 (9.94)	**0.002**
2-h insulin (pmol/L)	58.45 (61.78)	58.62 (50.51)	61.66 (60.51)	64.77 (62.21)	0.103
Fasting glucose (mmol/L)	5.39 (0.65)	5.42 (0.65)	5.51 (0.8)	5.58 (0.88)	** < 0.001**
2-h glucose (mmol/L)	5.79 (1.58)	5.68 (1.56)	5.89 (1.64)	6.00 (1.86)	**0.001**
Triglycerides (mmol/L)	1.11 (0.6)	1.16 (0.65)	1.26 (0.7)	1.32 (0.83)	** < 0.001**
Total HDL cholesterol ratio	3.48 (0.98)	3.52 (1)	3.63 (1.04)	3.69 (1.06)	** < 0.001**
LDL/HDL cholesterol ratio	2.26 (0.89)	2.30 (0.91)	2.40 (0.94)	2.44 (0.96)	** < 0.001**

Quartiles were defined by three cut points that divided the participants into four equally sized groups based on the standard deviations of bedtimes over seven days. Values are mean (standard deviation) unless otherwise stated. Numbers do not match owing to missing values

*PA* physical activity; *SBP* systolic blood pressure; *DBP* diastolic blood pressure; *BMI* body mass index; and *WC* waist circumference

^*^Heavy alcohol drinkers: men ≥ 40 g/day; women ≥ 20 g/day

**Table 2:** Characteristics of the 3,698 middle-aged birth cohort participants according to the quartiles of time in bed regularity (standard deviations of accelerometer-measured time in bed over seven days)

	Time in bed regularity (hh:mm:ss)				
	Q1 ≤ 0:54:55	Q2 0:54:56–1:16:12	Q3 1:16:13–1:43:17	Q4 ≥ 01:43:18	*p-value*
*n* (total = 3698)	925	924	925	924	
Factors related to sleep and time in bed					
Bedtime (hh:mm)	23:10 (1:02)	23:13 (1:01)	23:20 (1:09)	23:40 (1:34)	** < 0.001**
Wake-up time (hh:mm)	7:07 (1:01)	7:11 (1:02)	7:17 (1:05)	7:34 (1:25)	** < 0.001**
Time in bed (hh:mm)	7:56 (0:50)	7:58 (0:54)	7:58 (0:59)	7:54 (1:10)	0.198
Chronotype (*n* [%])					** < 0.001**
Morning-type	409 (46)	390 (44.7)	335 (38.2)	331 (38.3)	** < 0.05**
Day-type	444 (49.9)	452 (58)	497 (56.7)	449 (51.9)	** < 0.05**
Evening-type	37 (4.2)	31 (3.6)	45 (5.1)	85 (9.8)	** < 0.05**
Work schedule (*n* [%])					** < 0.001**
Day work	698 (75.5)	650 (70.3)	591 (52.8)	488 (52.8)	** < 0.05**
Shift work	89 (9.6)	119 (12.9)	156 (16.9)	245 (26.5)	** < 0.05**
Not working/no information available	138 (14.9)	155 (16.8)	178 (19.2)	191 (20.7)	** < 0.05**
Sociodemographic and lifestyle factors					
Men (*n* [%])	343 (37.2)	364 (39.6)	331 (35.8)	411 (44.6)	**0.001**
High education (*n* [%])	286 (32.6)	257 (30.3)	242 (28.0)	212 (24.7)	**0.002**
Current smoker (*n* [%])	125 (14)	131 (15)	163 (18.5)	201 (23.1)	** < 0.001**
Heavy alcohol drinkers* (*n* [%])	55 (5.9)	56 (6.1)	67 (7.2)	96 (10.4	**0.001**
Total PA (MET min/day)	1153.4 (353.3)	1097.5 (327.0)	1081.0 (315.5)	1015.5 (923.0)	** < 0.001**
Sedentary time (min)	571.7 (90.7)	578.9 (88.7)	575.5 (87.6)	582.0 (91.3)	0.131
Cardiometabolic health markers					
WC (cm)	89.2 (12.9)	90.0 (13.3)	91.0 (13.8)	92.6 (13.8)	** < 0.001**
Men	95.0 (11.4)	95.8 (11.4)	98.0 (12,6)	98.1 (12.5)	0.058
Women	85,2 (12.1)	86.2 (13.1)	87.2 (12.9)	88.2 (13.3)	** < 0.001**
BMI (kg/m^2^)	26 (4.7)	26.4 (4.8)	27 (5)	27.2 (5)	** < 0.001**
SBP (mmHg)	123.2 (15.7)	125.2 (15.2)	125 (15.6)	125.6 (16.3)	**0.010**
DBP (mmHg)	83 (10.5)	84.3 (10.2)	84.8 (10.4)	85.2 (10.9)	** < 0.001**
Body fat (%)	28.4 (9.0)	28.8 (9.4)	29.96 (9.3)	29.4 (9.5)	**0.003**
Fat mass (kg)	22.0 (10.0)	22.6 (10.8)	23.8 (11.3)	23.8 (11.2)	**0.001**
Visceral fat area (cm^2^)	100.0 (40.1)	102.4 (40.4)	107.2 (43.2)	108.2 (42.7)	** < 0.001**
Fasting insulin (pmol/L)	9.15 (6.56)	9.04 (5.91)	9.96 (10.4)	10.26 (8.68)	**0.007**
2-h insulin (pmol/L)	57.07 (53.03)	58.06 (55.33)	63.93 (66.29)	64.32 (60)	**0.027**
Fasting glucose (mmol/L)	5.43 (0.7)	5.46 (0.8)	5.49 (0.84)	5.52 (0.64)	**0.003**
2-h glucose (mmol/L)	5.76 (1.64)	5.74 (1.63)	5.88 (1.58)	5.96 (1.8)	**0.019**
Triglycerides (mmol/L)	1.14 (0.69)	1.16 (0.7)	1.25 (0.9)	1.28 (0.81)	** < 0.001**
Total HDL cholesterol ratio	3.51 (0.98)	3.57 (1.06)	3.64 (1.02)	3.61 (1.03)	**0.036**
LDL/HDL cholesterol ratio	2.29 (0.9)	2.34 (0.96)	2.41 (0.93)	2.37 (0.92)	**0.033**

Quartiles were defined by three cut points that divided the participants into four equally sized groups based on the standard deviations of time in bed over seven days. Values are mean (standard deviation) unless otherwise stated. Numbers do not match owing to missing values

*PA* physical activity; *SBP* systolic blood pressure; *DBP* diastolic blood pressure; *BMI* body mass index; and *WC* waist circumference

^*^Heavy alcohol drinkers: men ≥ 40 g/day; women ≥ 20 g/day

Analyses of differences between the quartiles showed that there was a significant inverse association between education and sleep–wake -rhythm regularity. The proportion of day shift workers was highest among those with the lowest bedtime, wake-up time, and TIB variability, while the proportion of shift workers was highest among those with the highest bedtime, wake-up time, and TIB variabilities. Smoking and heavy alcohol consumption were significantly related to higher variabilities in bedtime, wake-up time, and TIB (p < 0.05). Total PA was negatively associated with bedtime, wake-up time, and TIB regularities. Participants with the lowest bedtime, wake-up time, and TIB variabilities (Q1 in Tables 1, 2, and 3) had higher mean PA totals than participants in all other quartiles (Q2—Q4; post hoc analyses [all p < 0.05]; Tables 1, 2, and 3).

There were significant differences in the studied cardiometabolic health markers across the quartiles of bedtime, wake-up time, and TIB regularities (Tables 1, 2, and 3). Body mass index was higher among the participants with the highest variabilities in bedtime, wake-up time, and TIB than the participants with the lowest variabilities [post hoc analyses all (p < 0.001)]. Body fat was positively associated with wake-up time and TIB regularities but not with bedtime regularity (p = 0.203). The highest mean visceral fat area (111.4 cm2, SD = 45.4) was observed in the participants with the highest wake-up time variability (Q4 in Table 3), while the lowest mean visceral fat area (98.8 cm^2^, SD = 39.1) was remarked in the participants with the lowest bedtime variability (Q1 in Table 1).

**Table 3:** Characteristics of the 3,698 middle-aged birth cohort participants according to the quartiles of wake-up time regularity (standard deviations of accelerometer-measured wake-up time over seven days)

	Wake-up time regularity (hh:mm:ss)				
	Q1 ≤ 0:49:45	Q2 0:49:46–1:12:21	Q3 1:12:22–1:40:21	Q4 ≥ 01:40:22	*p-value*
*n* (total = 3698)	925	924	925	924	
Factors related to sleep and time in bed					
Bedtime (hh:mm)	22:59 (1:01)	23:10 (1:03)	23:20 (1:02)	23:53 (1:32)	** < 0.001**
Wake-up time (hh:mm)	7:03 (1:03)	7:14 (1:00)	7:15 (1:05)	7:37 (1:25)	** < 0.001**
Time in bed (hh:mm)	8:04 (0:56)	8:04 (1:00)	7:55 (0:54)	7:43 (1:06)	** < 0.001**
Chronotype (*n* [%])					** < 0.001**
Morning-type	449 (51)	383 (43.5)	343 (38.9)	290 (33.6)	** < 0.05**
Day-type	402 (45.7)	456 (51.8)	496 (56.3)	488 (56.5)	** < 0.05**
Evening-type	29 (3.3)	41 (4.7)	42 (4.8)	86 (10)	** < 0.05**
Work schedule (*n* [%])					** < 0.001**
Day work	655 (70.8)	649 (70.2)	643 (69.5)	480 (51.9)	** < 0.05**
Shift work	91 (9.8)	112 (12.1)	144 (15.6)	262 (28.4)	** < 0.05**
Not working/no information available	179 (19.4)	163 (17.6)	138 (14.9)	182 (19.7)	** < 0.05**
Sociodemographic and lifestyle factors					
Men (*n* [%])	373 (40.5)	329 (35.7)	368 (39.8)	379 (41.1)	**0.078**
High education (*n* [%])	283 (32.6)	284 (33.0)	241 (27.7)	189 (22.1)	** < 0.001**
Current smoker (*n* [%])	117 (13.2)	114 (13.1)	156 (17.6)	233 (26.8)	** < 0.001**
Heavy alcohol drinkers* (*n* [%])	55 (5.9)	58 (6.3)	65 (7.0)	96 (10.4)	**0.001**
Total PA (MET min/day)	1136.9 (366.0)	1072.7 (316.0)	1087.2(324.6)	1050.5 (327.9)	** < 0.001**
Sedentary time (min)	565.4 (93.8)	577.8 (82.3)	580.5 (91.5)	584.4 (89.6)	** < 0.001**
Cardiometabolic health markers					
WC (cm)	89.4 (12.9)	89.8 (13.5)	90.4 (12.9)	93.2 (14.4)	** < 0.001**
Men	95.0 (11.8)	96.6 (11.5)	97.2 (11.5)	99.1 (13.0)	**0.001**
Women	85.6 (12.2)	86.1 (13.0)	86.0 (11.9)	89.0 (14.0)	** < 0.001**
BMI (kg/m^2^)	26.3 (4.8)	26.5 (4.9)	26.5 (4.5)	27.4 (5.4)	** < 0.001**
SBP (mmHg)	123.8 (15.7)	124.1 (15.3)	124.8 (15.3)	126.3 (16.2)	**0.002**
DBP (mmHg)	83.5 (10.3)	83.7 (10.9)	84.6 (10.1)	85.6 (10.7)	** < 0.001**
Body fat (%)	28.3 (9.2)	29.1 (9.2)	29.0 (8.9)	30.2 (9.8)	**0.001**
Fat mass (kg)	22.0 (10.2)	22.8 (10.7)	22.7 (10.0)	24.7 (12.)	** < 0.001**
Visceral fat area (cm^2^)	100.4 (39.9)	102.9 (41.4)	103.1 (39.2)	111.4 (45.4)	** < 0.001**
Fasting insulin (pmol/L)	9.15 (6.77)	9.35 (7.58)	9.59 (7.0)	10.3 (10.46)	0.055
2-h insulin (pmol/L)	56.46 (55.89)	58.27 (52.34)	62.28 (65.2)	66.24 (60.95)	**0.001**
Fasting glucose (mmol/L)	5.42 (0.62)	5.48 (0.97)	5.48 (0.74)	5.52 (0.61)	**0.003**
2-h glucose (mmol/L)	5.74 (1.62)	5.74 (1.6)	5.85 (1.68)	6.02 (1.75)	**0.003**
Triglycerides (mmol/L)	1.15 (0.65)	1.18 (0.72)	1.2 (0.85)	1.31 (0.87)	** < 0.001**
Total HDL cholesterol ratio	3.58 (1.03)	3.53 (1.01)	3.56 (0.97)	3.65 (1.08)	0.152
LDL/HDL cholesterol ratio	2.35 (0.94)	2.31 (0.91)	2.35 (0.9)	2.4 (0.97)	0.247

Quartiles were defined by three cut points that divided the participants into four equally sized groups based on the standard deviations of wake-up times over seven days. Values are mean (standard deviation) unless otherwise stated. Numbers do not match owing to missing values

*PA* physical activity; *SBP* systolic blood pressure; *DBP* diastolic blood pressure; *BMI* body mass index; and *WC* waist circumference

^*^Heavy alcohol drinkers: men ≥ 40 g/day; women ≥ 20 g/day

Following the exclusion of shift workers (n = 609), the results remained the same with a few exceptions. When shift workers were excluded, there were significant differences in the mean values of fasting insulin between wake-up time quartiles (p = 0.034) and the mean values of 2-h insulin between bedtime quartiles (p = 0.047). The differences in the mean values of SBP across wake-up time regularity quartiles were no longer significant when shift workers were excluded from the analysis (p = 0.088). The mean values of 2-h insulin, total HDL cholesterol ratio, and LDL/HDL cholesterol ratio were not significant following the exclusion of shift workers across the TIB regularity quartiles.

In the unadjusted linear regression analyses, excepting SBP, which was not associated with wake-up time variability (p = 0.140), all cardiometabolic health markers were significantly and linearly associated with bedtime, wake-up time and TIB regularity variables. The health markers significantly associated with bedtime, wake-up time, and TIB regularities according to multivariate linear regression analyses are presented in Table 4, 5, and 6.

**Table 4:** Associations between cardiometabolic health markers and bedtime regularity at midlife in a population-based birth cohort (*N* = 3,698) according to multivariate linear regression analyses (Regression estimates for cardiometabolic health markers displaying a U-shaped relationship with wake-up time regularity are presented separately, stratified by ≤ 01:00:00 and > 01:00:00.)

Cardiometabolic health markers		B [95% CI]	*p-value*
Markers displaying a linear relationship with bedtime regularity			
WC (cm)	Model 1	**0.352 [0.197. 0.508]**	**< 0.001**
	Model 2A	**0.294 [0.136, 0.451]**	**< 0.001**
	Model 2B	**0.199 [0.042, 0.356]**	**0.013**
BMI (kg/m^2^)	Model 1	**0.239 [0.119. 0.359]**	**< 0.001**
	Model 2A	**0.194 [0.072, 0.316]**	**0.002**
	Model 2B	0.118 [− 0.003, 0.239]	0.057
SBP (mmHg)	Model 1	0.133 [− 0.037. 0.304]	0.124
	Model 2A	0.135 [− 0.035, 0.305]	0.121
	Model 2B	0.106 [− 0.063, 0.274]	0.219
DBP (mmHg)	Model 1	**0.189 [0.025. 0.354]**	**0.024**
	Model 2A	0.159 [− 0.006, 0.324]	0.059
	Model 2B	0.085 [− 0.079, 0.248]	0.312
Fasting insulin (mmol/L)	Model 1	**0.042 [0.006. 0.078]**	**0.022**
	Model 2A	0.028 [− 0.008, 0.065]	0.128
	Model 2B	0.005 [− 0.032, 0.041]	0.796
2-h insulin (mmol/L)	Model 1	**0.038 [0.009. 0.067]**	**0.010**
	Model 2A	0.024 [− 0.005, 0.054]	0.108
	Model 2B	0.001 [− 0.029, 0.03]	0.960
Fasting glucose (mmol/L)	Model 1	**0.259 [0.061. 0.457]**	**0.010**
	Model 2A	**0.224 [0.026, 0.422]**	**0.027**
	Model 2B	0.160 [− 0.037, 0.356]	0.112
2-h glucose (mmol/L)	Model 1	**0.088 [0.005. 0.171]**	**0.038**
	Model 2A	0.017 [− 0.066, 0.1]	0.683
	Model 2B	0.06 [− 0.023, 0.144]	0.158
Triglycerides (mmol/L)	Model 1	**0.072 [0.027. 0.116]**	**0.002**
	Model 2A	**0.053 [0.008, 0.099]**	**0.021**
	Model 2B	0.024 [− 0.021, 0.069]	0.298
Total HDL cholesterol ratio	Model 1	0.073 [− 0.007. 0.153]	0.075
	Model 2A	0.041 [− 0.040, 0.122]	0.325
	Model 2B	− 0.002 [− 0.083, 0.078]	0.953
Markers displaying U-shaped relationship with bedtime regularity			
LDL/HDL cholesterol ratio	Model 1	− 0.011 [− 0.056. 0.033]	0.617
Bedtime regularity of ≤ 01:00:00	Model 2A	− 0.022 [− 0.067, 0.023]	0.344
	Model 2B	− 0.033 [− 0.078, 0.012]	0.154
LDL/HDL cholesterol ratio	Model 1	0.004 [− 0.044. 0.052]	0.872
Bedtime regularity of > 01:00:00	Model 2A	− 0.008 [− 0.057, 0.04]	0.733
	Model 2B	0.003 [− 0.045, 0.052]	0.897
Body fat (%)	Model 1	**0.068 [0.01. 0.127]**	**0.023**
Bedtime regularity of ≤ 01:00:00	Model 2A	0.054 [− 0.006, 0.114]	0.076
	Model 2B	0.027 [− 0.034, 0.088]	0.379
Body fat (%)	Model 1	0.011 [− 0.050. 0.073]	0.719
Bedtime regularity of > 01:00:00	Model 2A	0.007 [− 0.057, 0.071]	0.820
	Model 2B	− 0.026 [− 0.091, 0.039]	0.433
Fat mass (kg)	Model 1	**0.052 [0.012. 0.091]**	**0.010**
Bedtime regularity of ≤ 01:00:00	Model 2A	**0.044 [0.004, 0.084]**	**0.033**
	Model 2B	0.03 [− 0.011, 0.07]	0.149
Fat mass (kg)	Model 1	0.002 [− 0.04. 0.043]	0.940
Bedtime regularity of > 01:00:00	Model 2A	0 [− 0.043, 0.043]	0.994
	Model 2B	− 0.019 [− 0.062, 0.024]	0.376
Visceral fat area (cm^2^)	Model 1	**0.045 [0.005. 0.085]**	**0.029**
Bedtime regularity of ≤ 01:00:00	Model 2A	0.037 [− 0.003, 0.078]	0.071
	Model 2B	0.022 [− 0.019. 0.063]	0.285
Visceral fat area (cm^2^)	Model 1	− 0.001 [− 0.045. 0.044]	0.981
Bedtime regularity of > 01:00:00	Model 2A	− 0.002 [− 0.048, 0.044]	0.926
	Model 2B	− 0.023 [− 0.069, 0.023]	0.322

Model 1 was adjusted for gender, education, marital status, work schedule, smoking status, alcohol risk use, time in bed, chronotype and medication (blood pressure/diabetes/lipids/medication affecting the central nervous system)

Model 2A: Model 1 further adjusted for sedentary time

Model 2B: Model 1 further adjusted for total physical activity

Significant results (p < 0.05) are indicated in bold

**Table 5:** Associations between cardiometabolic health markers and wake-up time regularity at midlife in a population-based birth cohort (*N* = 3,698) according to multivariate linear regression analyses (Regression estimates for cardiometabolic health markers displaying a U-shaped relationship with wake-up time regularity are presented separately, stratified by ≤ 01:00:00 and > 01:00:00.)

Cardiometabolic health markers		B [95% CI]	*p-value*	
Markers displaying a linear relationship wake− up time regularity				
BMI (kg/m^2^)	Model 1	**0.143 [0.032. 0.254]**	**0.012**	
	Model 2A	0.102 [− 0.011, 0.215]	0.076	
	Model 2B	0.047 [− 0.065, 0.160]	0.41	
SBP (mmHg)	Model 1	0.090 [− 0.067. 0.248]	0.261	
	Model 2A	0.086 [− 0.072, 0.243]	0.286	
	Model 2B	0.064 [− 0.092, 0.220]	0.423	
DBP (mmHg)	Model 1	**0.181 [0.029. 0.333]**	**0.020**	
	Model 2A	0.150 [− 0.002, 0.303]	0.054	
	Model 2B	0.097 [− 0.055, 0.249]	0.213	
Fasting insulin (mmol/L)	Model 1	**0.034 [0.001. 0.067]**	**0.046**	
	Model 2A	0.021 [− 0.012. 0.055]	0.210	
	Model 2B	0.005 [− 0.029. 0.038]	0.787	
2− h insulin (mmol/L)	Model 1	**0.049 [0.023. 0.076]**	** < 0.001**	
	Model 2A	**0.039 [0.012. 0.066]**	**0.005**	
	Model 2B	0.022 [− 0.005. 0.049]	0.114	
2− h glucose (mmol/L)	Model 1	**0.129 [0.053. 0.205]**	**0.001**	
	Model 2A	**0.107 [0.03. 0.184]**	**0.006**	
	Model 2B	0.076 [− 0.001. 0.153]	0.052	
Triglycerides (mmol/L)	Model 1	**0.067 [0.025. 0.108]**	**0.002**	
	Model 2A	**0.051 [0.009. 0.093]**	**0.017**	
	Model 2B	0.031 [− 0.011. 0.073]	0.152	
Total/HDL cholesterol ratio	Model 1	0.018 [− 0.056. 0.093]	0.627	
	Model 2A	− 0.011 [− 0.086. 0.064]	0.776	
	Model 2B	− 0.041 [− 0.116. 0.034]	0.280	
LDL/HDL cholesterol ratio	Model 1	0.009 [− 0.040. 0.059]	0.706	
	Model 2A	− 0.008 [− 0.057. 0.042]	0.756	
	Model 2B	− 0.026 [− 0.075. 0.023]	0.303	
Body fat (%)	Model 1	**0.081 [0.018. 0.145]**	**0.012**	
	Model 2A	0.049 [− 0.017, 0.114]	0.144	
	Model 2B	− 0.001 [− 0.067, 0.066]	0.979	
Markers displaying U− shaped relationship with wake− up time regularity				
WC (cm)	Model 1	0.129 [− 0.064. 0.321]	0.19	
Wake− up time regularity ≤ 1:00:00	Model 2A	− 0.067 [− 0.257, 0.124]	0.492	
	Model 2B	− 0.081 [− 0.272, 0.109]	0.401	
WC (cm)	Model 1	**0.214 [0.015. 0.413]**	**0.035**	
Wake− up time regularity > 1:00:00	Model 2A	**0.120 [0.019, 0.22]**	**0.019**	
	Model 2B	0.08 [− 0.021, 0.181]	0.118	
Fasting glucose (mmol/L)	Model 1	0.213 [− 0.036. 0.463]	0.094	
Wake− up time regularity of ≤ 1:00:00	Model 2A	0.021 [− 0.204, 0.247]	0.852	
	Model 2B	0 [− 0.225, 0.226]	0.997	
Fasting glucose (mmol/L)	Model 1	0.080 [− 0.168. 0.328]	0.525	
Wake− up time regularity of > 1:00:00	Model 2A	− 0.011 [− 0.140, 0.119]	0.872	
	Model 2B	− 0.035 [− 0.164, 0.094]	0.597	
Fat mass (kg)	Model 1	0.037 [− 0.021. 0.095]	0.210	
Wake− up time regularity of ≤ 1:00:00	Model 2A	− 0.013 [− 0.069, 0.043]	0.649	
	Model 2B	− 0.023 [− 0.08, 0.033]	0.415	
Fat mass (kg)	Model 1	0.048 [− 0.012. 0.107]	0.115	
Wake− up time regularity of > 1:00:00	Model 2A	0.022 [− 0.008, 0.053]	0.153	
	Model 2B	0.009 [− 0.022, 0.039]	0.581	
Visceral fat area (cm^2^)	Model 1	0.029 [− 0.03. 0.088]	0.335	
Wake− up time regularity of ≤ 1:00:00	Model 2A	− 0.021 [− 0.077, 0.035]	0.466	
	Model 2B	− 0.035 [− 0.092, 0.022]	0.233	
Visceral fat area (cm^2^)	Model 1	0.043 [− 0.021. 0.106]	0.188	
Wake− up time regularity of > 1:00:00	Model 2A	0.025 [− 0.007, 0.057]	0.125	
	Model 2B	0.011 [− 0.022, 0.044]	0.507	

Model 1 was adjusted for gender, education, marital status, work schedule, smoking status, alcohol risk use, time in bed, chronotype and medication (blood pressure/diabetes/lipids/medication affecting the central nervous system)

Model 2A: Model 1 further adjusted for sedentary time

Model 2B: Model 1 further adjusted for total physical activity

Significant results (p < 0.05) are indicated in bold

**Table 6:** Associations between cardiometabolic health markers and time in bed (TIB) regularity at midlife in a population-based birth cohort (*N* = 3698) according to multivariate linear regression analyses

Cardiometabolic health markers		B [95% CI]	* p-value*
Markers displaying a linear relationship time in bed regularity			
WC (cm)	Model 1	**0.223 [0.1. 0.346]**	**< 0.001**
	Model 2A	**0.196 [0.071, 0.321]**	**0.002**
	Model 2B	0.111 [− 0.014, 0.235]	0.082
BMI (kg/m^2^)	Model 1	**0.131 [0.036. 0.226]**	**0.007**
	Model 2A	**0.110 [0.013, 0.207]**	**0.026**
	Model 2B	0.042 [− 0.054, 0.138]	0.394
SBP (mmHg)	Model 1	0.107 [− 0.028. 0.242]	0.120
	Model 2A	0.114 [− 0.021, 0.249]	0.099
	Model 2B	0.090 [− 0.043, 0.224]	0.185
DBP (mmHg)	Model 1	**0.183 [0.053. 0.314]**	**0.006**
	Model 2A	**0.175 [0.044, 0.306]**	**0.009**
	Model 2B	0.112 [− 0.018, 0.242]	0.091
Fasting insulin (mmol/L)	Model 1	**0.039 [0.011. 0.068]**	**0.006**
	Model 2A	**0.035 [0.006. 0.064]**	**0.017**
	Model 2B	0.014 [− 0.014. 0.043]	0.328
2− h insulin (mmol/L)	Model 1	**0.037 [0.015. 0.06]**	**0.001**
	Model 2A	**0.033 [0.01. 0.056]**	**0.005**
	Model 2B	0.012 [− 0.011. 0.036]	0.295
Fasting glucose (mmol/L)	Model 1	**0.158 [0.002. 0.315]**	**0.048**
	Model 2A	0.090 [− 0.065. 0.246]	0.255
	Model 2B	0.147 [− 0.01. 0.304]	0.067
2− h glucose (mmol/L)	Model 1	**0.091 [0.025. 0.156]**	**0.007**
	Model 2A	**0.080 [0.014. 0.146]**	**0.018**
	Model 2B	0.042 [− 0.023. 0.108]	0.206
Triglycerides (mmol/L)	Model 1	**0.057 [0.022. 0.092]**	**0.002**
	Model 2A	**0.049 [0.013. 0.085]**	**0.008**
	Model 2B	0.023 [− 0.013. 0.059]	0.210
Total/HDL cholesterol ratio	Model 1	0.046 [− 0.018. 0.109]	0.158
	Model 2A	0.031 [− 0.033. 0.096]	0.341
	Model 2B	− 0.008 [− 0.072. 0.056]	0.800
LDL/HDL cholesterol ratio	Model 1	0.030 [− 0.012. 0.072]	0.159
	Model 2A	0.022 [− 0.021. 0.064]	0.314
	Model 2B	− 0.002 [− 0.044. 0.04]	0.938
Body fat (%)	Model 1	**0.080 [0.025. 0.134]**	**0.004**
	Model 2A	**0.064 [0.008, 0.120]**	**0.026**
	Model 2B	0.004 [− 0.053, 0.060]	0.902
Fat mass (kg)	Model 1	**0.055 [0.018. 0.092]**	**0.003**
	Model 2A	**0.046 [0.008, 0.083]**	**0.018**
	Model 2B	0.013 [− 0.025, 0.051]	0.496
Visceral fat area (cm^2^)	Model 1	**0.057 [0.019. 0.095]**	**0.003**
	Model 2A	**0.048 [0.009, 0.087]**	**0.017**
	Model 2B	0.014 [− 0.023, 0.053]	0.497

Model 1 was adjusted for gender, education, marital status, work schedule, smoking status, alcohol risk use, time in bed, chronotype and medication (blood pressure/diabetes/lipids/medication affecting the central nervous system)

Model 2A: Model 1 further adjusted for sedentary time

Model 2B: Model 1 further adjusted for total physical activity

Significant results (p < 0.05) are indicated in bold

Higher bedtime variability was associated with higher WC after adjustment for sedentary time, sleep-related factors, and other potential confounders [Model 2A: adjusted B 0.294, 95% CI (0.136, 0.451), p =  < 0.001] and after adjustment for total PA and other potential confounders (Model 2B: 0.199, 95% CI [0.042, 0.356], p = 0.013). After controlling for SED and other potential confounders (Model 2A), we observed a positive linear association between TIB variability and WC [0.196, 95% CI (0.071, 0.321), p = 0.002] and between wake-up time variability and WC in those with > 1-h wake-up time variability [0.120, 95% CI (0.019, 0.22), p = 0.019; Tables 5 and 6].

Following adjustment for SED and other potential confounders, higher bedtime and TIB variabilities were associated with higher BMIs (bedtime regularity: p = 0.002; TIB regularity: p = 0.006; Model 2A). Higher bedtime, wake-up time, and TIB variabilities were associated with higher triglyceride levels (bedtime regularity: adjusted B 0.053, 95% CI [0.008, 0.099], p = 0.021; wake-up time regularity: 0.051, 95% CI [0.009, 0.093], p = 0.017; TIB regularity: 0.049, 95% CI [0.013, 0.085], p = 0.008). Higher bedtime variability was associated with higher fasting glucose levels (0.224, 95% CI [0.026, 0.422], p = 0.027). Higher wake-up time and TIB variabilities were associated with higher 2-h insulin levels (p = 0.005 for both). Higher TIB variability was associated with higher fasting insulin and 2-h glucose levels (Table 6).

## Discussion

This study is the first one to examine the associations between device-estimated bedtime, wake-up time, and time in bed regularities and cardiometabolic health markers, including blood pressure level; abdominal adiposity level; and blood glucose, insulin, and cholesterol levels among middle-aged people. Higher bedtime variability was associated with higher WC regardless of time in bed, chronotype, and sedentary time or total PA. Even after adjustment for time in bed, chronotype, and sedentary time, higher bedtime, wake-up time, and TIB variabilities were associated with poorer glucose and insulin regulation and higher triglyceride levels. However, when we considered total PA rather than sedentary time in our analyses, several cardiometabolic health markers were no longer associated with sleep regularity.

Our study results support earlier findings that sleep irregularity is a risk factor for poor cardiometabolic health [17, 18, 50, 51]. In addition, our results align with previous findings suggesting that physical activity supports cardiometabolic health [27]. Behind the association between sleep and activity behaviors could be a virtuous circle: A person who sleeps well is more energetic and active during the day [52], and in turn, this person’s daytime activity supports their improved sleep quality at night [22, 26] and facilitates circadian alignment [24, 25]. Evidence has suggested that poor sleep quality increases sedentary behavior [22, 52] and is associated with reduced appetite control and unhealthy dietary habits [22], which are risk factors for obesity. In addition, having inconsistent bedtimes was associated with higher sedentary time in a population-based study [28]. In our study, sedentary time did not appear to modify the associations between bedtime, wake-up time, and TIB regularities and cardiometabolic health markers in the same way that physical activity did (Table 6).

The results of the present study suggest that physical activity may have a beneficial impact on cardiometabolic health, even though sleep rhythms may not consistently support this notion. This phenomenon might be attributed to the strong associations between physical activity and both physical and mental well-being. For example, stress has been linked to the development of cardiovascular diseases [53], but physical activity can play a significant role in stress management [54]. Additionally, physical activity can enhance various physiological functions and metabolism, such as increasing insulin sensitivity [55]. Furthermore, physical activity contributes to achieving deeper and more restful sleep, making it easier to fall asleep and reducing sleep latency, while also potentially preventing nighttime awakenings [56, 57]. However, the timing of physical activity appears to be relevant to cardiometabolic health [58], with evening physical activity potentially adversely influencing sleep quality [59]. In the present study, it was observed that participants with irregular bedtimes had higher WC than participants with regular bedtimes, despite controlling for total levels of physical activity, sedentary time, and other potential confounders. These findings encourage further investigation of the role of 24-h activity rhythm for cardiometabolic health.

It has already been well established that morning light exposure advances the circadian phase, but that evening light exposure suppresses melatonin secretion, thereby delaying the circadian phase [60]. In fact, consistent wake time is a key part in insomnia treatment to support good sleep [28, 49]. In addition to light exposure, rest–activity rhythms and feeding schedules are important time cues that synchronize the human circadian system with the environment, regulate metabolic pathways, and, thus, actively contribute to humans’ metabolic regulation systems [61]. In practice, always getting out of bed at the same time could also support regular light exposure and breakfast times. Similarly, when a person regularly goes to bed at the same time, their eating window from breakfast to evening snack is likely also regularly scheduled at the same time within the 24-h timeframe. Evidence has suggested that regular eating patterns are associated with cardiometabolic health [62]. To conclude, regular sleep rhythm within the 24-h timeframe enables regular lifestyle also while awake, including eating window or timing of light exposure and physical activity, and thereby may support cardiometabolic health as well.

The strength of this large-scale, population-based study of sleep regularity and cardiometabolic health lies in its focus on all three regularity variables, namely bedtime, wake-up time, and time in bed regularities, and also on daytime activity behaviors. Additionally, this study focuses on the general population and represents all economic and occupational sectors rather than limiting itself to shift workers. This study considers a wide range of cardiometabolic health markers, including blood pressure level; abdominal adiposity level; and blood glucose, insulin, and cholesterol levels. The study protocol was the same for all participants, and the participants did not receive any feedback from the activity monitors. We used device-based measurements of one week’s bedtime regularity, wake-up time regularity, TIB regularity, total PA, and sedentary time to potentially better estimate 24-h activity behaviors (i.e., sleep [63], total PA, and sedentary time [64]). Additionally, the device-based measurements considered both weekend and weekday data, which were not analyzed separately. Based on our knowledge, this is the first study examining separately device-based wake-up time regularity and bedtime and time in bed regularities among middle-age population.

This study also has some limitations. The study sample was homogenous regarding age and ethnicity. The methodological limitations of accelerometer-estimated sleep schedules should be acknowledged. Although accelerometry data gathered over one week have been widely used in sleep regularity studies, a longer data period would better represent daily sleep patterns [65]. Additionally, despite the upward trend in cardiometabolic health markers, the participants were rather healthy. Therefore, the clinical significance of the results requires further study. Also, the statistical limitations of having a large number of analyses should be acknowledged. Because this study used a cross-sectional design, the causality of the associations with cardiometabolic health markers could not be determined. We considered a comprehensive set of potential confounders that have been highlighted in previous literature, such as chronotype, time in bed, and work schedule. Still, information about diet, eating schedules, and energy intake were not available [61, 66, 67]. Therefore, we were unable to consider participants' dietary habits, fasting schedules, and energy intake, which have been previously established as factors associated with cardiometabolic and circadian health.

In conclusion, this population-based study of middle-aged adults revealed consistent positive associations between bedtime, wake-up time, and time in bed irregularities and cardiometabolic health markers following adjustment for sedentary time and other potential risk factors such as chronotype, time in bed, work schedule. However, these positive associations turned non-significance after adjustments for physical activity and other potential risk factors. The findings suggest that daytime physical activity can be one facilitating factor in maintaining cardiometabolic health in an irregular sleep rhythm. In addition, the role of 24-h activity rhythm in cardiometabolic health warrant further investigation.

## Supplementary Information

**Additional file 1.** Satterplots with a quadratic curve between cardiometabolic health markers which showed to have U-shaped relationship between 7-day SD of bedtime and 7-day SD of wake-up time in data from 3,698 middle-aged birth cohort participants.

**Additional file 2: Table S1.** Characteristics of the 3698 middle-aged birth cohort participants according to quartiles of midpoint of sleep regularity (SD of accelerometer-measured midpoint of sleep across seven days).

**Additional file 3: Table S2.** Pearson correlation coefficient (PCC) values for bedtime, wake-up time, and time in bed regularity values for totally 25,886 nights from 3698 participants from a population-based birth cohort.

## Data Availability

NFBC data is available from the University of Oulu, Infrastructure for Population Studies. Permission to use the data can be applied for research purposes via an electronic material request portal. In the use of data, we follow the EU general data protection regulation (679/2016) and Finnish Data Protection Act. The use of personal data is based on cohort participant’s written informed consent at his/her latest follow-up study, which may cause limitations to its use. Please, contact NFBC project center (NFBCprojectcenter (at) oulu.fi) and visit the cohort website for more information.

## Abbreviations

TIB: Time in bed
PA: Physical activity
SBP: Systolic blood pressure
DBP: Diastolic blood pressure
BMI: Body mass index
WC: Waist circumference
