# Supplementary material for: Regularity of bedtime, wake-up time, and time in bed in mid-life: associations with cardiometabolic health markers with adjustment for physical activity and sedentary time
Source: J Act Sedentary Sleep Behav. 2024 Jan 5;3:2. doi: 10.1186/s44167-023-00040-6 (PMC11960235; doi:10.1186/s44167-023-00040-6)

Additional file 3. Satterplots with a quadratic curve between cardiometabolic health markers which showed to have U-shaped relationship between 7-day SD of bedtime in data from 3,698 middle-aged birth cohort participants.


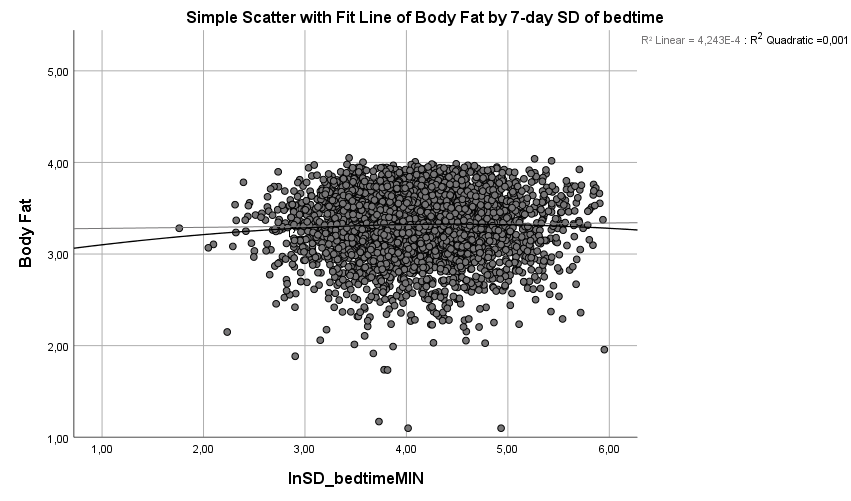


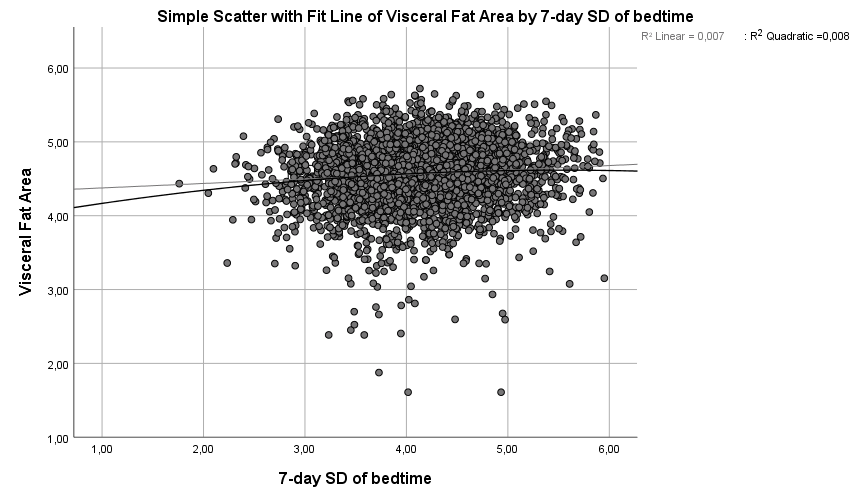


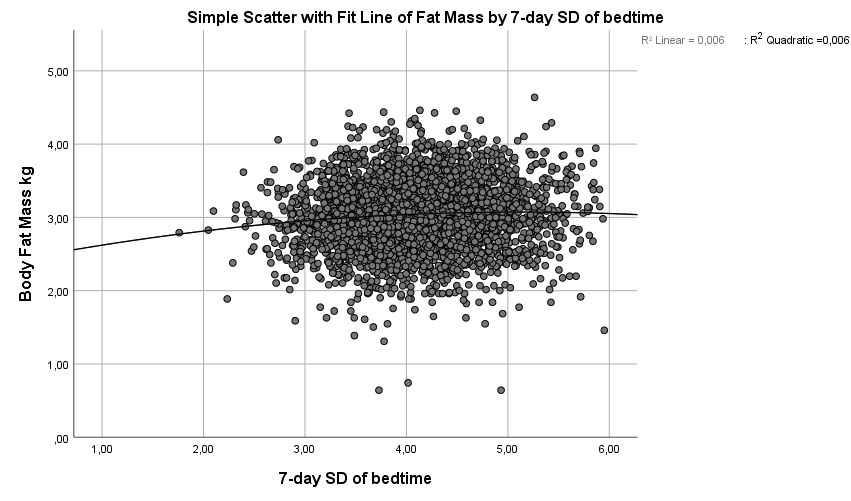


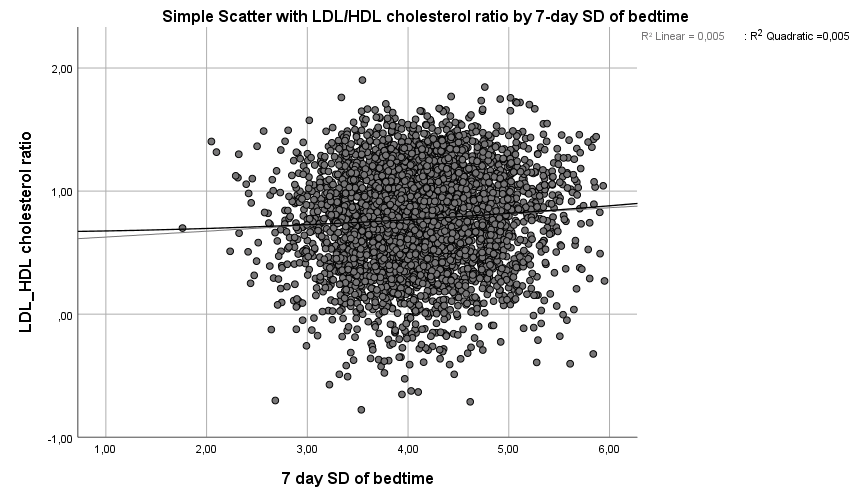


**Suppl. Material.** Scatterplots with a quadratic curve between cardiometabolic health markers which showed to have U-shaped relationship between 7-day SD of wake-up time in data from 3,698 middle-aged birth cohort participants.


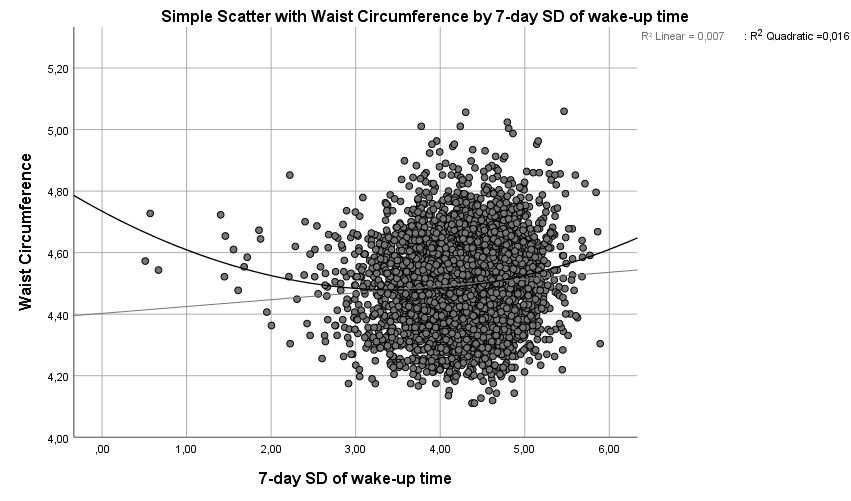


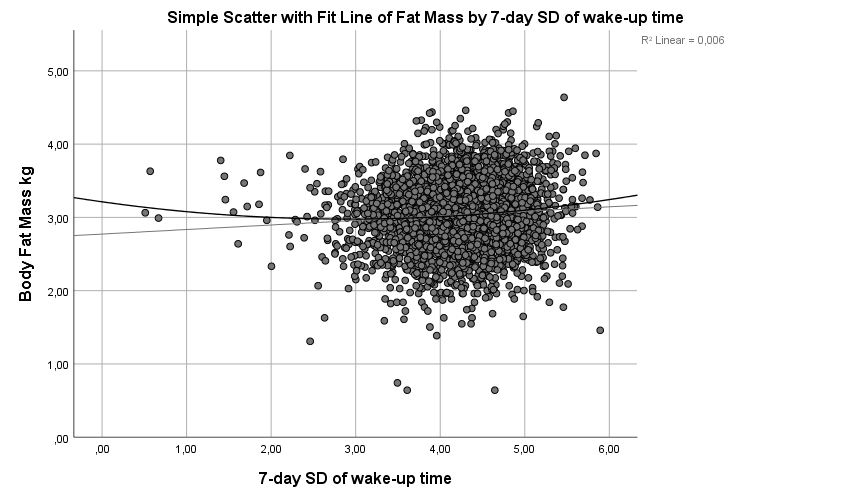


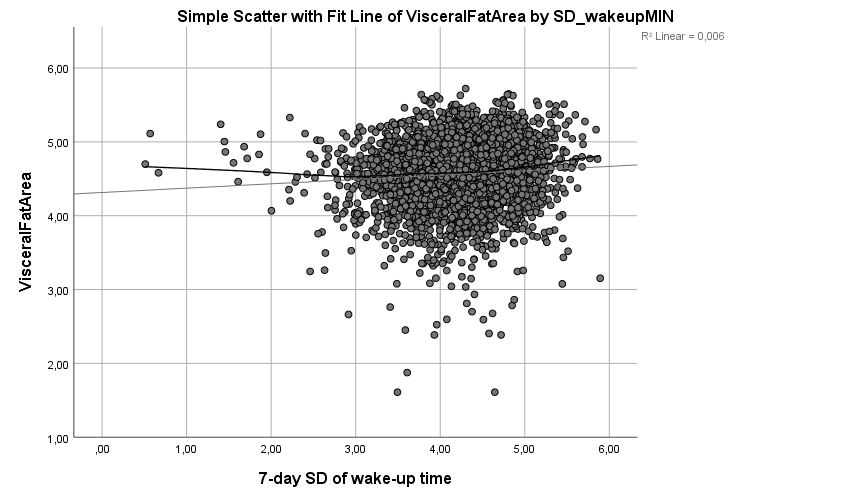


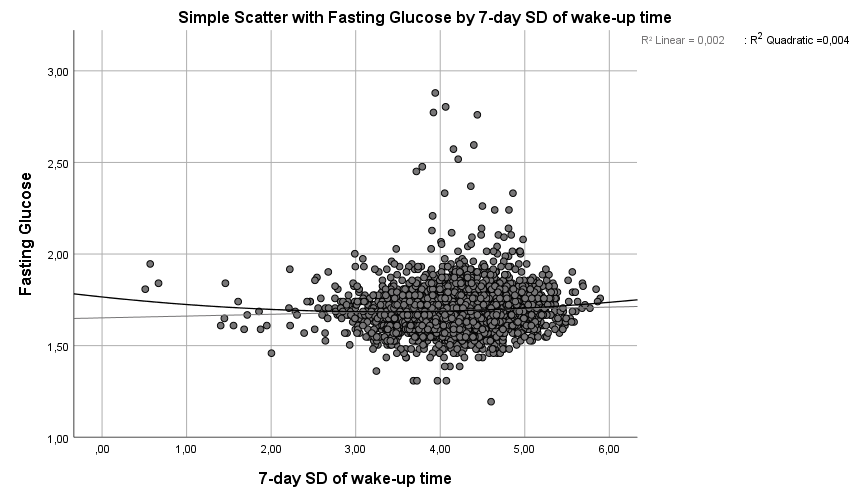

Supplement: Supplementary file 1 — Additional file 1. Satterplots with a quadratic curve between cardiometabolic health markers which showed to have U-shaped relationship between 7-day SD of bedtime and 7-day SD of wake-up time in data from 3,698 middle-aged birth cohort participants. [file 44167_2023_40_MOESM1_ESM.docx]
