# Supplementary material for: Regularity of bedtime, wake-up time, and time in bed in mid-life: associations with cardiometabolic health markers with adjustment for physical activity and sedentary time
Source: J Act Sedentary Sleep Behav. 2024 Jan 5;3:2. doi: 10.1186/s44167-023-00040-6 (PMC11960235; doi:10.1186/s44167-023-00040-6)
Supplement: Supplementary file 2 — Additional file 2: Table S1. Characteristics of the 3698 middle-aged birth cohort participants according to quartiles of midpoint of sleep regularity (SD of accelerometer-measured midpoint of sleep across seven days). [file 44167_2023_40_MOESM2_ESM.docx]

Table S1. Characteristics of the 3698 middle-aged birth cohort participants according to quartiles of midpoint of sleep regularity (SD of accelerometer-measured midpoint of sleep across seven days).

|  | | | **Midpoint of sleep regularity (hh:mm:ss)** | | | |  |
| --- | --- | --- | --- | --- | --- | --- | --- |
|  | | | **Q1**  **≤0:39:07** | **Q2**  **0:39:08 – 00:55:03** | **Q3**  **00:55:04 – 01:18:59** | **Q4**  **≥01:19:00** |  |
| *n* (total = 3,698) | | | 925 | 924 | 925 | 924 |  |
| Factors related to sleep and time in bed | | | | | | | *p-value* |
| Bedtime (hh:mm) | | | 23:10 (1:02) | 23:13 (1:01) | 23:20 (1:09) | 23:40 (1:34) | **< 0.001** |
| Wake-up time (hh:mm) | | | 7:07 (1:01) | 7:11 (1:02) | 7:17 (1:05) | 7:34 (1:25) | **< 0.001** |
| Time in bed (hh:mm) | | | 7:56 (0:50) | 7:58 (0:54) | 7:58 (0:59) | 7:54 (1:10) | 0.198 |
| Chronotype (*n* [%]) | | | | | | | |
|  | Morning type | | 434 (48.7) | 351 (39.9) | 360 (41.5) | 320 (36.9) | **< 0.05** |
|  | Day type | | 426 (47.8) | 480 (54.6) | 467 (53.8) | 469 (54.1) | **< 0.05** |
|  | Evening type | | 31 (3.5) | 48 (5.5) | 41 (4.7) | 78 (9) | **< 0.05** |
| Work schedule (*n* [%]) | | |  |  |  |  |  |
|  | Day shift | | 643 (69.5) | 650 (70.3) | 642 (69.4) | 492 (53.2) | **< 0.05** |
|  | Other shift | | 100 (10.8) | 1242 (13.4) | 139 (15.0) | 246 (26.6) | **< 0.05** |
|  | Not working/no information available | | 182 (19.7) | 150 (16.2) | 144 (15.6) | 186 (20.1) | **< 0.05** |
| Sociodemographic and lifestyle factors | | | | | | | *p-value* |
| Men (*n* [%]) | | | 338 (36.7) | 363 (39.4) | 332 (34.8) | 426 (46.2) | **<0.05** |
| High education (*n* [%]) | | | 287 (32.8) | 274 (31.8) | 247 (28.9) | 189 (22.0) | **< 0.05** |
| Current smoker (*n* [%]) | | | 107 (12.0) | 107 (12.1) | 156 (17.8) | 250 (28.7) | **< 0.05** |
| Heavy alcohol drinkers* (*n* [%]) | | | 46 (5.0) | 52 (5.6) | 69 (7.5) | 107 (11.6) | **< 0.05** |
| Total PA (MET min/day) | | | 1148.4 (364.9) | 1088.4 (327.5) | 1056.4 (307.4) | 1054.2 (331.7) | **< 0.001** |
| Sedentary time (min) | | | 562.4 (93.5) | 575.3 (87.7) | 581.8 (84.5) | 588.7 (90.6) | **< 0.001** |
| Cardiometabolic health markers | | | | | | | *p-value* |
| WC (cm) | | | 88.6 (12.9) | 90.1 (12.9) | 90.9 (13.7) | 93.2 (14.2) | **< 0.001** |
|  | | Men | 95.0 (11.4) | 96.0 (11.1) | 97.7 (12.6) | 98.8 (12.6) | **< 0.001** |
|  | | Women | 84.9 (12.4) | 86.3 (12.5) | 87.3 (12.8) | 88.3 (13.6) | **< 0.001** |
| BMI (kg/m^2^) | | | 26 (4.8) | 26.5 (4.6) | 26.8 (5) | 27.2 (5.2) | **< 0.001** |
| SBP (mmHg) | | | 123.2 (15.4) | 125 (15.9) | 124.1 (15.1) | 126.6 (16.3) | **<0.001** |
| DBP (mmHg) | | | 83 (10.3) | 84.4 (10.5) | 84.3 (10.5) | 85.6 (10.6) | **<0.001** |
| Body fat (%) | | | 28.28 (9.06) | 28.89 (9.21) | 30.09 (9.33) | 29.36 (9.54) | **0.001** |
| Fat mass (kg) | | | 21.72 (10.15) | 22.6 (10.34) | 23.83 (11.17) | 23.95 (11.51) | **< 0.001** |
| Visceral fat area (cm^2^) | | | 99.0 (39.7) | 102.3 (40.0) | 107.4 (43.0) | 109.0 (43.3) | **< 0.001** |
| Fasting insulin (pmol/L) | | | 8.96 (6.08) | 9.44 (8.13) | 9.71 (8.04) | 10.29 (9.71) | **0.039** |
| 2-h insulin (pmol/L) | | | 55.64 (53.1) | 58.36 (59.51) | 63.58 (60.87) | 65.82 (61.49) | **<0.001** |
| Fasting glucose (mmol/L) | | | 5.39 (0.67) | 5.45 (0.65) | 5.51 (0.96) | 5.55 (0.67) | **< 0.001** |
| 2-h glucose (mmol/L) | | | 5.69 (1.57) | 5.73 (1.52) | 5.88 (1.66) | 6.06 (1.87) | **0.001** |
| Triglycerides (mmol/L) | | | 1.12 (0.61) | 1.19 (0.73) | 1.22 (0.92) | 1.32 (0.82) | **< 0.001** |
| Total HDL cholesterol ratio | | | 3.51 (1.02) | 3.58 (1.01) | 3.55 (1.00) | 3.68 (1.06) | **0.003** |
| LDL/HDL cholesterol ratio | | | 2.29 (0.93) | 2.34 (0.9) | 2.34 (0.91) | 2.43 (0.97) | **0.012** |
| *Note:* Quartiles were defined by three cut points that divided the participants into four equally sized groups based on the standard deviations of time in bed over seven days. Values are mean standard deviations unless otherwise stated. Numbers do not match owing to missing values.  Abbreviations: PA = physical activity; SBP = systolic blood pressure; DBP = diastolic blood pressure; BMI = body mass index; and WC = waist circumference.  *Heavy alcohol drinkers: men ≥40 g/day; women ≥20 g/day. | | | | | | | |
