# Supplementary material for: Regularity of bedtime, wake-up time, and time in bed in mid-life: associations with cardiometabolic health markers with adjustment for physical activity and sedentary time
Source: J Act Sedentary Sleep Behav. 2024 Jan 5;3:2. doi: 10.1186/s44167-023-00040-6 (PMC11960235; doi:10.1186/s44167-023-00040-6)
Supplement: Supplementary file 3 — Additional file 3: Table S2. Pearson correlation coefficient (PCC) values for bedtime, wake-up time, and time in bed regularity values for totally 25,886 nights from 3698 participants from a population-based birth cohort. [file 44167_2023_40_MOESM3_ESM.docx]

Table S2. Pearson correlation coefficient (PCC) values for bedtime, wake-up time, and time in bed regularity values for totally 25,886 nights from 3,698 participants from a population-based birth cohort.

|  | **PCC*** | | | |
| --- | --- | --- | --- | --- |
| **Measure** | **Bedtime regularity** | **Wake-up time regularity** | **Time in bed regularity** | **Midpoint of sleep regularity** |
| **Bedtime regularity** | 1 | 0.42 | 0.51 | 0.79 |
| **Wake-up time regularity** | 0.42 | 1 | 0.55 | 0.78 |
| **Time in bed regularity** | 0.51 | 0.55 | 1 | 0.48 |
| **Midpoint of sleep regularity** | 0.79 | 0.78 | 0.48 | 1 |

*All PCC p-values <0.001
